# Editorial: Bioinformatics in the age of data science: algorithms, methods, and tools applied from Omics to structural data

**DOI:** 10.3389/fbinf.2023.1246859

**Published:** 2023-07-04

**Authors:** Diego Mariano, Neli José Da Fonseca Júnior, Lucianna Helene Santos, Raquel Cardoso de Melo-Minardi

**Affiliations:** ^1^ Laboratory of Bioinformatics and Systems, Universidade Federal de Minas Gerais, Belo Horizonte, Brazil; ^2^ European Molecular Biology Laboratory, European Bioinformatics Institute (EMBL-EBI), Wellcome Genome Campus, Hinxton, Cambridgeshire, United Kingdom; ^3^ Biomolecular Simulations Group, Institut Pasteur de Montevideo, Montevideo, Uruguay

**Keywords:** bioinformatics, web tools, algorithms, methods, structural bioinformatics, omics sciences, software

In recent years, researchers have been confronted with an overwhelming influx of data, resulting from novel technologies utilized for sequencing data and acquiring three-dimensional structures of macromolecules. The increasing demand for computing power has allowed an evolution of data processing technologies, ranging from using CPUs with increasingly miniaturized lithography to the adaptation of graphics cards for efficient data analysis. As a result, we live in a new era: the age of data science.

We extended an open invitation to researchers and general software developers to disclose and share their methods, algorithms, and tools with the community of bioinformatics and computational biology in a Research Topic entitled “Bioinformatics in the age of data science: algorithms, methods, and tools applied from Omics to structural data”.

We received four submissions: two data reports (Rodrigues et al.; Martins et al.), one technology and code (Mohsen et al.), and one brief research report (Oliveira et al.). Thus, this Research Topic contains a combination of articles describing new algorithms, methods, and tools that are applicable to the domains of bioinformatics and computational biology. Below, we summarize the contributions that comprise this Research Topic.

In the data report section, we received two remarkable contributions that yielded significant works in terms of source code and user-friendly tools. Rodrigues et al. presented an application for the analysis of multi-omic bacterial genomes entitled “PanViTa: Pan Virulence and resisTance analysis.”. PanViTa (Pan Virulence and resisTance Analysis) was developed in Python, accessible at https://github.com/dlnrodrigues/panvita.

Martins et al. presented an update for the public database entitled “Propedia v2.3: A novel representation approach for the peptide-protein interaction database using graph-based structural signatures (Martins et al.)”. Propedia is a public database of three-dimensional protein–peptide complexes, and it is available at http://bioinfo.dcc.ufmg.br/propedia2. In this new version, Propedia received an increment of approximately 150% in the number of protein–peptide complexes available (from ∼19,800 to ∼49,300). Additionally, the authors provided graph-based structural signature data for subsets of sequence-based and complex-type data. These signature vectors can be used in several machine-learning tasks, such as protein–peptide docking prediction.

In the technology and code section, Mohsen et al. presented a Snakemake pipeline for QIIME2 16S data analysis, called Snaq, in the paper “Snaq: A Dynamic Snakemake Pipeline for Microbiome Data Analysis With QIIME2.”. Snaq is available at https://github.com/attayeb/snaq.

In the brief research report section, Oliveira et al. presented a tool called SPLACE in their article “SPLACE: A tool to automatically SPLit, Align, and ConcatenatE genes for phylogenomic inference of several organisms.” The authors described SPLACE as a tool to split, align, and concatenate the genes automatically and then generate a supermatrix file and phylogenetic tree. SPLACE is available at https://github.com/reinator/splace.

These recently developed software programs are accessible to the public or offered as open source, thereby enabling the scientific community to benefit from them. All the software programs presented here display easy-to-access graphical interfaces, sometimes considered essential for most users, and comprehensive documentation and availability of tutorials.

In summary, this Research Topic highlights important contributions to the field of bioinformatics and computational biology. We hope the works presented here can give insights into and inspiration for developing new tools and lead to new scientific discoveries.

